# MnO_2_ coated multi-layer nanoplatform for enhanced sonodynamic therapy and MR imaging of breast cancer

**DOI:** 10.3389/fbioe.2022.955127

**Published:** 2022-10-19

**Authors:** Yan Xu, Wanlin Tan, Mingyu Chen, Sijie Chen, Kui Tang, Haiqin Liao, Chengcheng Niu

**Affiliations:** ^1^ Department of Ultrasound Diagnosis, The Second Xiangya Hospital, Central South University, Changsha, Hunan, China; ^2^ Research Center of Ultrasonography, The Second Xiangya Hospital, Central South University, Changsha, Hunan, China

**Keywords:** sonodynamic therapy, magnetic resonance imaging, tumor hypoxia, reactive oxygen species, manganese dioxide

## Abstract

Sonodynamic therapy (SDT) is a promising new anti-tumor therapy that inhibits tumor growth by ultrasound activation of sonosensitizers to produce reactive oxygen species (ROS). However, the problems of hypoxia in the microenvironment within solid tumors and the effectiveness of SDT will decrease due to the little accumulation of sonosensitizers at the tumor site, as well as tumor cell tolerance, have limited the development of SDT. To overcome these problems, a core-shell structured nanoparticle (IR780/PLGA@MnO_2_ NPs) loaded with IR780 and manganese dioxide (MnO_2_) was developed as a nanocarrier to transport the sonosensitizer IR780 and the generated oxygen into the tumor tissue. The MnO_2_ shell layer of IR780/PLGA@MnO_2_ NPs can prevent the premature release of IR780 in the blood and also it can react with acidic and high H_2_O_2_, the generated oxygen can relieve tumor tissue hypoxia, and the generated Mn can enhance magnetic resonance imaging (MRI) signal intensity by acting as a contrast agent for MRI. More importantly, the released IR780 can produce ROS to kill tumor cells under ultrasound excitation. This PH-responsive and H_2_O_2_-triggered SDT based on the IR780/PLGA@MnO_2_NPs is an effective platform to inhibit tumor growth with negligible systemic toxicity. This work develops a multifunctional therapeutic integrated nanoplatform for breast cancer treatment, which is expected to be used in the clinic.

## Introduction

Advances in cancer diagnosis and therapy have attracted immense attention in achieving accurate treatment to minimize side effects and improving the survival rate of patients. Ultrasound (US), as a mechanical wave, is widely used in clinical US imaging for cancer diagnosis and high-intensity focused US for cancer therapy (Wu et al., 2005; Wells, 2006; Hussain and Nguyen, 2014). Moreover, US could facilitate both the drug release and cellular uptake by inducing reversible cell deformation and membrane permeabilization (Ahmadi et al., 2020). In recent years, sonodynamic therapy (SDT), based on US, as a non-invasive treatment in promoting malignant tumor deterioration, has led to burgeoning research interest, which has the distinct advantages of deep penetration, low cost and low toxicity (Wood and Sehgal, 2015; Qian et al., 2016; Wan et al., 2016). SDT employs activating sonosensitizers through US and utilizes oxygen molecules of tissues into toxic reactive oxygen species (ROS), which can oxidize crucial biomacromolecules such as proteins, enzymes, DNA of tumor cells, thus inhibiting the tumor growth (Rosenthal et al., 2004; Costley et al., 2015; Zhang et al., 2019; Son et al., 2020; Wang et al., 2020). However, the organic sonosensitizer molecule hinders its further application of SDT due to low bioavailability and biological instability (Son et al., 2020). To overcome these limitations, our previous studies have found that loading the organic sonosensitizer such as IR780 or ICG onto polymeric PLGA could effectively improve the biocompatibility and biological stability (Niu et al., 2017; Wang et al., 2018; Chen et al., 2020; Huang et al., 2020; Pei et al., 2020; Xu et al., 2020).

SDT progress requires enough oxygen molecules in the process of cancer cell destruction, however, the tumor hypoxic microenvironment significantly some inherent resistances to SDT due to the abnormal proliferation of cells, vascular abnormalities, and lymphatic system dysfunction (Hockel and Vaupel, 2001; Dong et al., 2021). Moreover, the high consumption of oxygen in the process of SDT can worsen the hypoxic microenvironment of tumor tissues and conversely influences the therapeutic efficacy of SDT (Wang et al., 2020; Dong et al., 2021). Therefore, how to overcome the limitation of hypoxia in the tumor area on the low ROS-generation efficacy of SDT is a challenging subject. Several strategies have developed to relieve hypoxia, such as using perfluorocarbon to deliver additional oxygen, inhaling supply hyperbaric oxygen, and catalyzing overexpressed hydrogen peroxide (H_2_O_2_) to O_2_ in the tumor tissues (Song et al., 2016a; Song et al., 2016b; Zhu et al., 2018; Huang et al., 2020). Manganese dioxide (MnO_2_) and its various nanocomposites, as a catalase-like nanoenzyme to product oxygen by the decomposition of excess H_2_O_2_ in tumor area is the most effective way to relieve hypoxia, have been attracting increasing attention (Chu et al., 2017; Zhu et al., 2018; Zhang et al., 2020; Zhang and Ji, 2020). Thus, utilizing tumor PH- and H_2_O_2_- sensitive MnO_2_ to relieve hypoxia is promising way, the process of MnO_2_ at mild acid and excess H_2_O_2_ condition can be expressed by the following reactions (Gordijo et al., 2015):

$${MnO}_{2}+H_{2}O_{2}+2H^{+}\to {Mn}^{2+}+H_{2}O+O_{2}$$

Many studies have already manifested that nanostructured MnO_2_ can be act as a catalyst to react with overexpressed H_2_O_2_ in tumor microenvironment to produce O_2_, which could surmount hypoxia in tumor area (Zhu et al., 2018; Yang et al., 2019; Liu et al., 2021). In addition, the process of nanostructured MnO_2_ can generate water-soluble Mn^2+^ ions which can be used as an excellent magnetic resonance imaging (MRI) contrast agent for early and accurate diagnosis of cancer (Huang et al., 2017; Liu et al., 2018; Fu et al., 2019; Ding et al., 2020). Furthermore, nanostructured MnO_2_ can also assemble anticancer drug molecules and release them when itself degrade in an acidic environment, which could be used as “gatekeeper” carrier for drug delivery (Ma et al., 2017).

**FIGURE 1:**
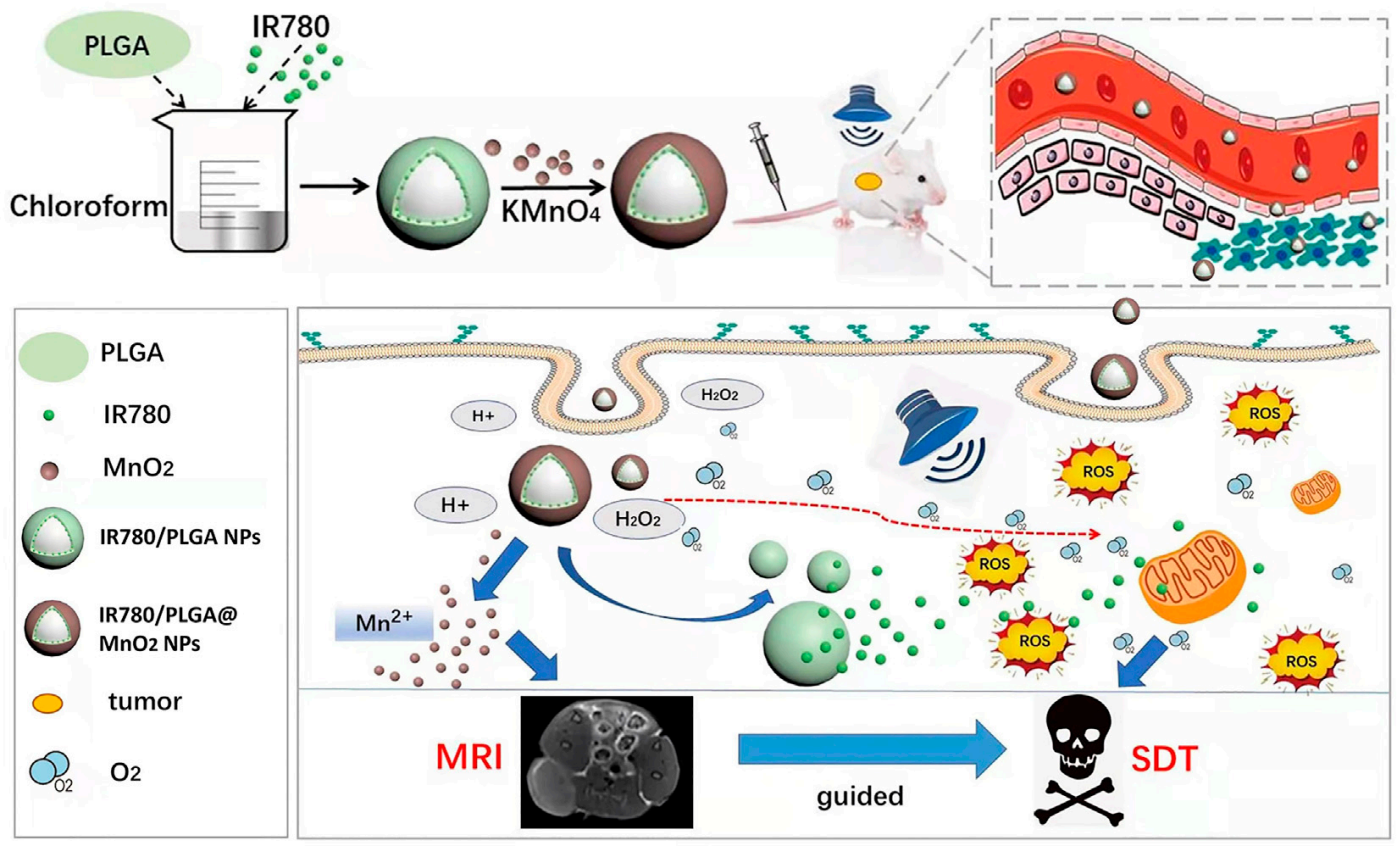
Schematic illustration of the IR780/PLGA@MnO_2_ NPs used for enhance SDT and MR imaging of breast cancer.

Therefore, we designed a core-shell structure multi-layer nanoparticle (IR780/PLGA@MnO_2_ NPs) as sonosensitizer carrier and the oxygen reservoir to enhance the efficacy of SDT and MR imaging (Figure 1). Meanwhile, the MnO_2_ shell layer of these IR780/PLGA@MnO_2_ NPs could prevent IR780 release in the blood circulation, while react with H^+^ and H_2_O_2_ in the tumor area, then decomposing and releasing the IR780 from IR780/PLGA@MnO_2_ NPs. Finally, the generation of oxygen could relieve tumor hypoxia, making the exhibit higher ROS generation efficiency even under hypoxia atmosphere, the generation of Mn^2+^ ions could act as the contrast agents for T1-weighted magnetic resonance imaging (MRI) for the recognition and diagnosis of tumor. Thus, the developed IR780/PLGA@MnO_2_ NPs would be a potential nanoplatform for controllable tumor oxygenation and MRI-guided tumor-growth suppression.

## Materials and methods

### Preparation and characterization of IR780/PLGA@MnO_2_ NPs

Firstly, 200 mg PLGA and 6 mg IR780 was fully dissolved in 6 ml chloroform, then the above mixture was put into 30 ml PVA (4%) solution, emulsified for 2 min using an ultrasonic processor (5 s on and 5 s off), 20 ml deionized water was added into the mixture and stirred for 12 h in order to volatilize the chloroform. Then, the resulting IR780/PLGA NPs was washed with deionized water at least 3 times. To form layered manganese oxide, the prepared IR780/PLGA NPs were dispersed in 100 ml deionized water, then 300 mg potassium permanganate was added, the resulting IR780/PLGA @MnO2 NPs was stirred for 6 h in dark place, washed with deionized water at least 3 times, and kept at 4°C in dark place.

The morphology, structure and element mapping of the IR780/PLGA@MnO_2_ NPs was detected by Transmission electron microscopy (TEM, Hitachi, Ltd., Japan), the size and zeta potential of the NPs were tested by dynamic light scattering (DLS, ZEN3600, Malvern Instruments, United Kingdom), and the sizes of the NPs in PBS and 10% fetal bovine serum (FBS) were tested for 7 days to study the size stability. The optical properties of various NPs (PLGA NPs, IR780/PLGA NPs, IR780/PLGA@MnO_2_ NPs) were tested by a UV-vis-NIR spectrophotometer (UV-3600, Shimadzu, Japan). The Mn content was calculated by ICP-OES (ICP-OES730, Agilent Co. Ltd., USA). The amount of IR780 encapsulated into the IR780/PLGA@MnO_2_ NPs were recorded by a UV-vis-NIR spectrophotometer.

### Oxygen Storage and IR780 Release from the IR780/PLGA@MnO_2_ NPs in Response to H_2_O_2_

A dissolved oxygen meter (AMT08, United States) was used to measure the O_2_ concentration of IR780/PLGA@MnO_2_ NPs in response to H_2_O_2_. Ten milliliters of deoxygenated water with H_2_O_2_ (25 µM), IR780/PLGA NPs or IR780/PLGA@MnO_2_ NPs were loaded into a glass bottle. The real-time O_2_ concentration in the bottle was recorded every 10 s, the IR780 release experiment of the IR780/PLGA@MnO_2_ NPs was recorded by a UV-vis-NIR spectrophotometer. The IR780/PLGA@MnO_2_ NPs were loaded into dialysis bags (1 ml) and put into different buffers, including PBS (pH 7.4) and PBS (pH 5.5) with 25 µM H_2_O_2_, PBS (pH 5.5) without 25 µM H_2_O_2_ to partially mimic the microenvironments of healthy tissue and tumor tissue. At the different time points up to 72 h, IR780 concentration of each time point was recorded according to the standard curve of IR780.

### Measurement of ROS generation *in vitro*

SOSG was used as a fluorescent probe to measure the production of ^1^O_2_, 1 ml of IR780/PLGA@MnO_2_ NPs (100 μg/ml) and 1 µL of (SOSG, 5 µM) was irradiated by US transducer at an ultrasonic intensity of 1 W/cm^2^ with the duty cycle of 40% in different time (0, 30, 60, 90, 120, 150 or 180 s). The SOSG fluorescence was tested by a fluorescence spectrometer (Tecan, Männedorf, Switzerland) for IR780/PLGA@MnO_2_ NPs, H_2_O_2_ and IR780/PLGA NPs, the relative efficiency of ^1^O_2_ production was calculated by F1 (under US irradiation)/F0 (before US irradiation).

### Intracellular uptake

4T1 mice breast cancer cells were cultured at 37°C in RPMI-1640 medium containing 10% (v/v) FBS, 1% (v/v) penicillin G and streptomycin, 4T1 cells (1 × 10^4^ cells/well) were seeded on laser confocal petri dish for 24 h. Then, DiI labeled IR780/PLGA@MnO_2_ NPs (200 μL, 1 mg/ml) were added and co-incubated for 0, 4, 12 and 24 h, washed for 3 times in dark, fixed with 4% paraformaldehyde for 15 min and stained with DAPI for 5 min. Finally, the cells were observed by a laser confocal microscope.

### Cellular reactive oxygen species generation

DCFH-DA cellular ROS assay kit was used to detect intracellular ROS production. 4T1 cells (1 × 10^5^ cells/well) were seeded in the 6-well plates and treated with different conditions: (Hussain and Nguyen, 2014) PBS, (Wells, 2006) US, (Wu et al., 2005) IR780/PLGA NPs, (Ahmadi et al., 2020) IR780/PLGA NPs + US, (Qian et al., 2016) IR780/PLGA@MnO_2_ NPs, (Wood and Sehgal, 2015) IR780/PLGA@MnO_2_ NPs + US. The intensity of US is 1.0 W/cm^2^ and the time is 2 min, the NPs concentration is 1.0 mg/ml and the dosage is 200 µL. After incubation for 24 h, DCFH-DA (10 µM) was added to each well, ROS production was determined by fluorescence microscopy.

### Cell viability assay

Mice breast cancer 4T1 cells and human breast cancer MCF-7 cells (5 × 10^3^ cells/well) were seeded in a 96-well plate and cultured overnight under normoxic or hypoxic conditions, respectively. To test the cell viabilities of different IR780/PLGA@MnO_2_ NPs concentrations, 50 µL of IR780/PLGA@MnO_2_ NPs with different concentrations (1.65, 3.25, 6.25, 12.5, 25.0, and 50.0 μg/ml) were added into the each well and incubated overnight, respectively, then the cell viability was calculated with a CCK-8 cytotoxicity assay kit. To test the cell viabilities of different treatments under normoxic or hypoxic conditions, cells were randomly divided into six groups: (Hussain and Nguyen, 2014) PBS, (Wells, 2006) US, (Wu et al., 2005) IR780/PLGA NPs, (Ahmadi et al., 2020) IR780/PLGA NPs + US, (Qian et al., 2016) IR780/PLGA@MnO_2_ NPs, (Wood and Sehgal, 2015) IR780/PLGA@MnO_2_ NPs + US. The intensity of US is 1.0 W/cm^2^, the time is 2 min The NPs concentration is 6.25 μg/ml and the dosage is 50 µL. After 24 h of incubation, the cell viability was determined using a CCK-8 cytotoxicity assay kit.

### Apoptosis assay

4T1 cells (1 × 10^5^ cells/well) were seeded in the 6-well plates and cultured for 24 h. The cells were treated with different conditions: (Hussain and Nguyen, 2014) PBS, (Wells, 2006) US, (Wu et al., 2005) IR780/PLGA NPs, (Ahmadi et al., 2020) IR780/PLGA NPs + US, (Qian et al., 2016) IR780/PLGA@MnO_2_ NPs, (Wood and Sehgal, 2015) IR780/PLGA@MnO_2_ NPs + US. The intensity of US is 1.0 W/cm^2^, the time is 2 min, the NPs concentration is 1.0 mg/ml and the dosage is 200 µL. Then the cells were washed 3 times and stained with PI and DAPI, and observed by fluorescence microscopy.

### Animal model

Female BALB/c mice (6 weeks old, 20 g) were obtained from the Hunan Silaike Laboratory Animal Corporation (Changsha, China). 4T1 cells (1 × 10^6^) were injected into the mice by subcutaneous injection to establish a breast tumor model when the tumor volume reached 80 mm^3^. All animal experiments were approved by the Ethics Committee of the Second Xiangya Hospital of Central South University and conducted in accordance with the guidelines of the Department of Laboratory Animals of Central South University.

### Biodistribution and biosafety

For evaluating the biodistribution of IR780/PLGA@MnO_2_ NPs, 15 tumor-bearing BALB/c mice (*n* = 3) were injected with 200 μL of IR780/PLGA@MnO_2_ NPs (1.0 mg/ml) *via* the tail vein (0, 4, 8, 12, 24 h before imaging) for *in vivo* fluorescent imaging (Ex: 745 nm, Em: 840 nm) by a Lumina IVIS Spectrum imaging system (PerkinElmer, United States). Mice of each group were sacrificed after the whole-body imaging, then the major organs and tumor were taken out for *in vitro* imaging and the average fluorescence intensity was further analyzed.

To further assess the biotoxicity of IR780/PLGA@MnO_2_ NPs *in vivo*, 10 tumor-bearing BALB/c mice (*n* = 5) were randomly divided into 2 groups: (Hussain and Nguyen, 2014) saline and (Wells, 2006) IR780/PLGA@MnO_2_ NPs. Mice in the two groups were injected with 200 μL of saline or IR780/PLGA@MnO_2_ NPs (2.0 mg/ml) *via* the tail vein, then the H&E stain of main organs was observed after 14 days injection.

### MR imaging *in vitro* and *in vivo*

MR imaging *in vitro* were tested on a clinical by MRI instrument (UR770 3.0T, United imaging Ltd., China) equipped with a small animal coil. Different MnO_2_ concentrations (Mn concentrations: 0.00, 0.05, 0.10, 0.20, 0.30, and 0.40 mM) of the IR780/PLGA@MnO2 NPs were dispersed in different media: (Hussain and Nguyen, 2014) pH 7.4; (Wells, 2006) pH 5.5, 25 µM H_2_O_2_. Then, the different concentrations of IR780/PLGA@MnO_2_ NPs were added to 2 ml Eppendorf tubes for MR imaging (T1 WI). The T1 relaxation time was measured, and the T1 relaxation coefficient (r1) was calculated.

In the *in vivo* T1-weighted MRI experiments, 200 μL of IR780/PLGA@MnO_2_ NPs solution (2 mg/ml) was intravenously injected into 4T1 tumor-bearing mice. The T1-weighted MR images of tumor sites were captured before and 24 h after injection of IR780/PLGA@MnO_2_ NPs. Finally, the SI within the ROI of the MR images were measured.

### *In vivo* antitumor effect

To investigate hypoxia environment *in vivo*, the 4T1 tumor-bearing mice (*n* = 5) were randomly divided with different treatments: (Hussain and Nguyen, 2014) saline, (Wells, 2006) IR780/PLGA NPs, (Wu et al., 2005) IR780/PLGA@MnO_2_ NPs. After 24 h of injection, immunofluorescence of an antibody against hypoxia-inducible factor-1α (HIF-1α) in the tumor was observed.

To investigate the antitumor effect *in vivo*, the 4T1 tumor-bearing mice (*n* = 5) were randomly divided with different treatments: (Hussain and Nguyen, 2014) saline, (Wells, 2006) US, (Wu et al., 2005) IR780/PLGA NPs, (Ahmadi et al., 2020) IR780/PLGA@MnO_2_ NPs, (Qian et al., 2016) IR780/PLGA NPs + US, (Wood and Sehgal, 2015) IR780/PLGA@MnO_2_ NPs + US, 200 µL of different NP solutions (2 mg/ml) was intravenously injected into the mice every 2 days. After injection for 8 h, the tumors of the mice in groups (Wells, 2006), (Qian et al., 2016), and (Wood and Sehgal, 2015) were irradiated with US (the ultrasonic intensity is 2 W/cm^2^ and duration is 10 min). Body weight and tumor volume were recorded every other day for 14 days. On day 15, the tumors of the mice were obtained. In addition, TUNEL and PCNA immunohistochemical staining were performed on the tumor tissues to observe tumor cell apoptosis and proliferation, respectively.

### Statistical analysis

The experimental data were presented as the mean ± SD. Comparisons of two groups were analyzed by Student’s t-test and multiple groups were analyzed by two-way analysis using GraphPad Prism 8 software. *p* < 0.05 was considered significant.

## Results and discussion

### Characterization of IR780/PLGA@MnO_2_ NPs

**FIGURE 2:**
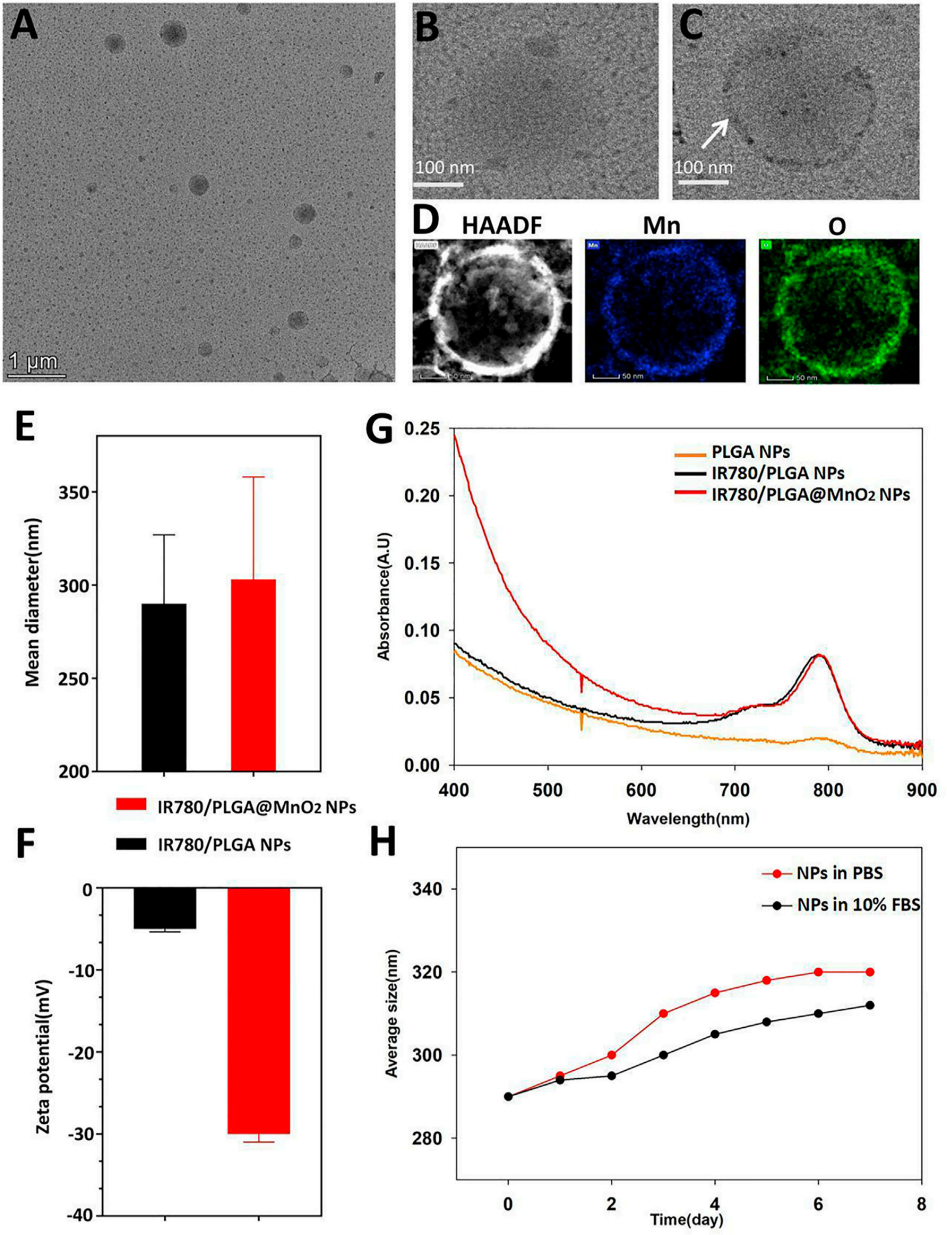
Characterization of IR780/PLGA@MnO_2_ NPs. **(A–C)** The TEM images of IR780/PLGA@MnO_2_ NPs **(A)** and IR780/PLGA NPs **(B)**, the white arrow indicated the MnO_2_ on the shell of IR780/PLGA@MnO_2_ NPs **(C)**. **(D)** The elemental mapping of Mn and O of IR780/PLGA@MnO_**2.**_ **(E)** Size and **(F)** zeta potential of IR780/PLGA NPs with and without MnO_2_ shell. **(G)** UV−vis−NIR absorption spectra of IR780/PLGA NPs, PLGA NPs, IR780/PLGA@MnO_2_ NPs. **(H)** Size distributions of IR780/PLGA@MnO_2_ NPs in PBS and 10% FBS for 7 days.

As shown in Figure 2B of the TEM image, spherical IR780/PLGA NPs nanoparticles were successfully constructed. The shell of IR780/PLGA@MnO_2_ NPs coating of MnO_2_ was proved by TEM images (Figures 2A,C). The elemental mapping of Mn and O of IR780/PLGA@MnO_2_ (Figure 2D). The size of the IR780/PLGA NPs was approximately 293.05 ± 5.43 nm and increased to 300.34 ± 4.56 nm after MnO_2_ coating (Figure 2E). The zeta potential decreased from −5.3269 mV to −30.563 mV after MnO_2_ coating, indicating that the IR780/PLGA@MnO_2_ NPs is more stable in the blood than the IR780/PLGA NPs (Figure 2F). The UV absorption spectra are shown in Figure 2G, there was an absorption peak at 780–790 nm of the IR780/PLGA@MnO_2_ NPs, which indicated that the PLGA/IR780@MnO_2_ NPs was successfully loaded the IR780. The stability of the IR780/PLGA@MnO_2_ NPs in PBS and 10% FBS solution were shown in Figure 2H. The entrapment efficiency of IR780 in the IR780/PLGA@MnO_2_ NPs was calculated to be 58.47% ± 1.48%. The Mn content in the IR780/PLGA@MnO_2_ NPs was approximately 0.2761 mg/ml.

### PH and H_2_O_2_ responsive IR780 release and responsive oxygen generation

**FIGURE 3:**
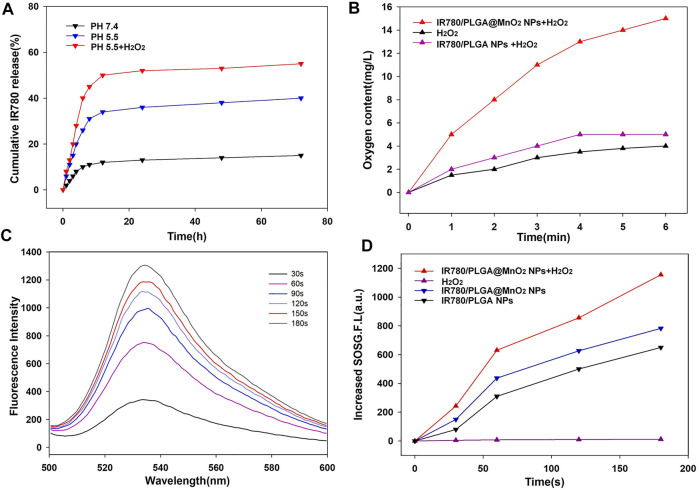
**(A)** The IR780 release from IR780/PLGA@MnO_2_ NPs at pH 7.4 or 5.5 with or without H_2_O_2_. **(B)** Oxygen content of IR780/PLGA MnO_2_ NPs with H_2_O_2_. **(C)** Time-dependent ^1^O_2_ generation of IR780/PLGA MnO_2_ NPs irradiated by US (1 MHz, 1.0 W/cm^2^). **(D)** Time-dependent fluorescence increasement of SOSG with different treatments under ultrasound irradiation.

It is known that tumor microenvironment is featured with regional hypoxia, low PH and increasing H_2_O_2_ level due to insufficient blood supplement. The MnO_2_ can react with the increasing H^+^ and H_2_O_2_ in the solid tumor to produce O_2_ and Mn^2+^ (Sun et al., 2021). When the IR780/PLGA@MnO_2_ NPs entered the tumor tissue, MnO_2_ shell gradually dissolved, the IR780/PLGA NPs core exposed, and IR780 release from the IR780/PLGA@MnO_2_ NPs in the tumor (Figure 3A). Compared with the IR780/PLGA@MnO_2_ NPs in PBS (pH 7.4), the IR780/PLGA@MnO_2_ NPs in PBS (pH 5.5) with 25 µM H_2_O_2_ exhibited considerably higher IR780 release, and the release rate of IR780 approached 32% within 72 h. For the IR780/PLGA@MnO_2_ NPs in PBS (pH 5.5) without 25 µM H_2_O_2_, the drug release rate was only 20%, revealing that the IR780 release rate was in H_2_O_2_ and PH-responsive way.

The oxygen release profiles of IR780/PLGA@MnO_2_ NPs were analyzed under hypoxic conditions (Figure 3B). While the IR780/PLGA@MnO_2_ NPs was dispersed in the deoxygenated medium containing H_2_O_2_, the oxygen concentration in water increased from 4 to 13 mg/L water in the first 1 min. Few O_2_ generation was observed while the IR780/PLGA@MnO_2_ NPs was dispersed in the deoxygenated medium without H_2_O_2_. The oxygen concentration of the IR780/PLGA@MnO_2_ NPs with H_2_O_2_ group was obviously higher than that in the IR780/PLGA@MnO_2_ NPs without H_2_O_2_ group all the time. It means that the oxygen generation the IR780/PLGA@MnO_2_ NPs with H_2_O_2_ was fast and continuous.

### Singlet oxygen generation capacity

Based on the efficient oxygen generation of MnO_2_, SOSG was used as a probe to measure the ^1^O_2_ production to assess the SDT effect of the IR780/PLGA@MnO_2_ NPs with US irradiation. As shown in Figure 3C, at a final IR780/PLGA@MnO_2_ NPs concentration of 100 μg/ml, the fluorescence intensity of SOSG increased along with the time of US irradiation (0, 60, 120, and 180 s), indicating that the ^1^O_2_ production capacity of the IR780/PLGA@MnO_2_ NPs is outstanding and have the potential against cancer through SDT. As shown in Figure 3D, the fluorescence intensity of SOSG was 819 at 180 s in the IR780/PLGA@MnO_2_ NPs without H_2_O_2_ group. Surprisingly, for the IR780/PLGA@MnO_2_ NPs with H_2_O_2_, the fluorescence intensity of SOSG was increased as high as 1150. Therefore, more ^1^O_2_ generation can be attributed to the increased oxygen concentration from MnO_2_ which react with H_2_O_2_ in the SDT process. These results demonstrated that the IR780/PLGA@MnO_2_ NPs can effectively generate ^1^O_2_ under US irradiation and can be used for sonodynamic treatment of cancer.

### Cell uptake and reactive oxygen species determination

**FIGURE 4:**
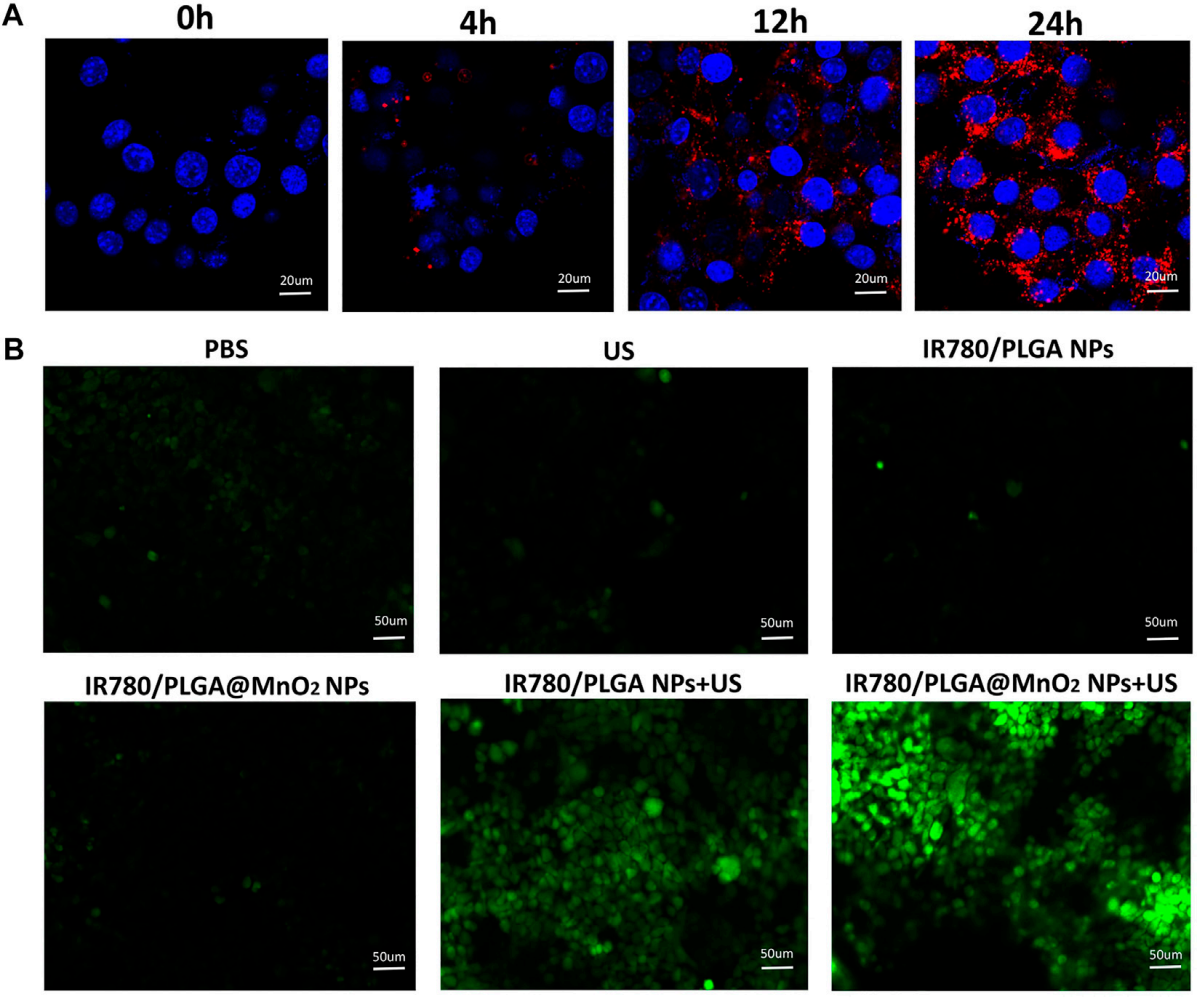
**(A)** Fluorescence images of 4T1 cells incubated with IR780/PLGA@MnO_2_ NPs for 0, 4, 12 and 24 h (scale bar: 20 μm). **(B)** Fluorescence microscope images of DCFH-DA stained 4T1 cells subjected to different treatments: (Hussain and Nguyen, 2014) PBS, (Wells, 2006) US, (Wu et al., 2005) IR780/PLGA NPs, (Ahmadi et al., 2020) IR780/PLGA@MnO_2_ NPs, (Qian et al., 2016) IR780/PLGA NPs + US, (Wood and Sehgal, 2015) IR780/PLGA@MnO_2_ + US (scale bar, 50 µm).

To understand the cell uptake of the IR780/PLGA@MnO_2_ NPs by 4T1 cells, DiI-labeled NPs was used to co-incubation with 4T1 cells for 0, 4, 12, and 24 h. As shown in Figure 4A, an obvious red fluorescence of the IR780/PLGA@MnO_2_ NPs was observed in 4 h and increased over time, which demonstrated that the longer co-incubation time between the IR780/PLGA@MnO_2_ NPs and cells, the more nanoparticles phagocytized by 4T1 cells.

To understand intracellular oxidative stress of the IR780/PLGA@MnO_2_ NPs to examine the sonodynamic activity, DCFH-DA was used as a probe. As shown in Figure 4B, barely green fluorescence was observed in (Hussain and Nguyen, 2014) PBS, (Wells, 2006) US, (Wu et al., 2005) IR780/PLGA NPs and (Ahmadi et al., 2020) IR780/PLGA@MnO_2_ groups, which suggested that only US irradiation or only NPs cannot produce ^1^O_2_. Obvious green fluorescence was observed in (Qian et al., 2016) IR780/PLGA NPs + US and (Wood and Sehgal, 2015) IR780/PLGA@MnO_2_ + US groups, which suggested both the NPs and US irradiation were necessary for generation of ^1^O_2_. Comparing between groups (Qian et al., 2016) and (Wood and Sehgal, 2015), the group of IR780/PLGA@MnO_2_ + US irradiation has stronger green fluorescence intensity than the group of IR780/PLGA NPs + US irradiation, which suggested that with the help of MnO_2_ shell reacting with H_2_O_2_, the IR780/PLGA@MnO_2_ NPs could generate more O_2_ and then product more ^1^O_2_. Therefore, with loading of the shell of MnO_2_ to provide O_2_, the IR780/PLGA@MnO_2_ NPs have excellent properties of SDT against cancer.

### *In vitro* biocompatibility and antitumor activity

**FIGURE 5:**
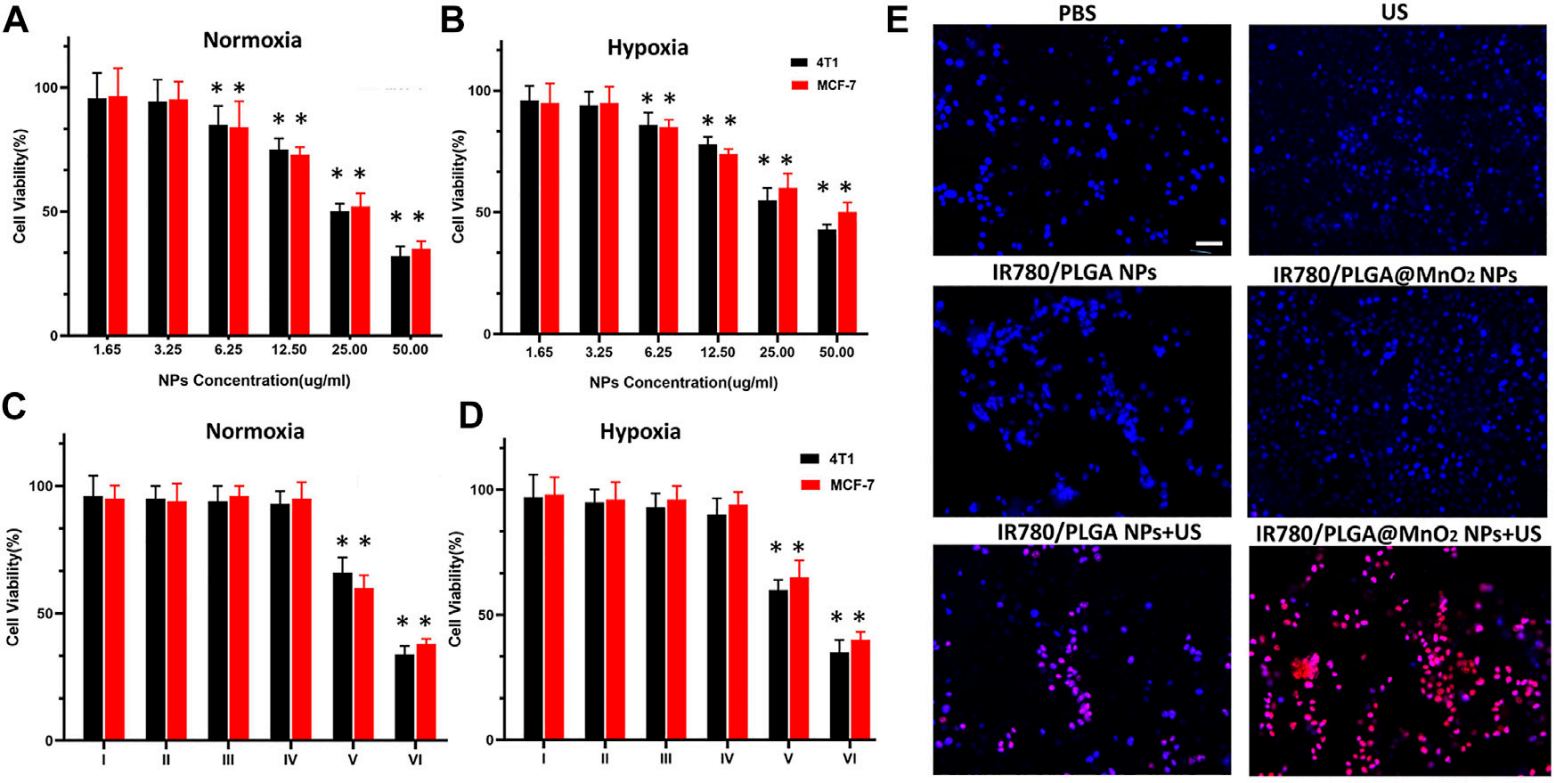
**(A,B)** Cell viability of 4T1 and MCF-7 cells incubated with different concentrations of IR780/PLGA@MnO2 NPs under normoxia environment **(A)** and hypoxia environment **(B)**. **(C,D)** Cell viability of 4T1 and MCF-7 cells under different treatments: (I) PBS, (II) US, (III) IR780/PLGA NPs, (IV) IR780/PLGA@MnO_2_, (V) IR780/PLGA NPs + US, (VI) IR780/PLGA@MnO_2_ + US) in normoxia environment **(C)** and hypoxia environment **(D)**. **(E)** Fluorescence microscope images of DAPI and PI co-stained 4T1 cells after various treatments: (Hussain and Nguyen, 2014) PBS, (Wells, 2006) US only, (Wu et al., 2005) IR780/PLGA NPs, (Ahmadi et al., 2020) IR780/PLGA@MnO_2_ NPs, (Qian et al., 2016) IR780/PLGA NPs + US, (Wood and Sehgal, 2015) IR780/PLGA@MnO_2_ + US (scale bar, 100 µm). (*p < 0.05, compared with the control group).

Cytotoxicity and antitumor activity of IR780/PLGA@MnO_2_ NPs under normoxic and hypoxic environments were measured in 4T1 cells and MCF-7 cells. According to CCK8 assay (Figures 5A,B), when the concentration of IR780/PLGA@MnO_2_ was 0–6.25 µg/ml, the cell survival rate of CCK8 was more than 95% under normoxic and hypoxic conditions, which suggested low toxicity of IR780/PLGA@MnO_2_ NPs, and 6.25 µg/ml is the appropriate concentration for further biological experiment. When the concentration increased, the cell survival rate of 4T1 cell and MCF-7 cells obviously decreased. To evaluate the antitumor activity of IR780/PLGA@MnO_2_ NPs through SDT, the 4T1 and MCF-7 viabilities in normoxic and hypoxic environments treated with different conditions was measured (Figures 5C,D). In the normoxic and hypoxic environments, both the 4T1 and MCF-7 cell viabilities of US group were up to 90%, indicating that US exposure (1W/cm^2^) is safe to cells. When the IR780/PLGA NPs and IR780/PLGA@MnO_2_ NPs combined with US exposure, the 4T1 cell viabilities further decreased. Treatment of IR780/PLGA@MnO_2_ NPs combined with US exposure results in 75% cell death, which was higher than that of IR780/PLGA NPs results in 45% cell death, suggesting that the MnO_2_ shell coated in IR780/PLGA@MnO_2_ NPs enhances more cell apoptosis through the SDT effects due to more O_2_ production.

A live/dead staining assay was used to evaluate the therapeutic efficiency of IR780/PLGA@MnO_2_ NPs under US irradiation. Blue fluorescence of DAPI stained with all cells and red fluorescence of PI stained with dead cells. As shown in Figure 5E, there is bare red fluorescence in the (Hussain and Nguyen, 2014) PBS, (Wells, 2006) US, (Wu et al., 2005) IR780/PLGA NPs and (Ahmadi et al., 2020) IR780/PLGA@MnO_2_ NPs groups. Red fluorescence was observed when the cells treated with IR780/PLGA NPs under US irradiation. Furthermore, more red fluorescence was observed in the IR780/PLGA@MnO_2_ NPs under US irradiation group, which further confirmed that with the help of MnO_2_ shell respective to H_2_O_2_ to produce O_2_, the SDT efficacy of IR780 is enhanced and suggested that IR780/PLGA@MnO_2_ NPs is an efficient sonodynamic agent for hypoxic cancer cells under US irradiation.

### Biodistribution and biosafety

**FIGURE 6:**
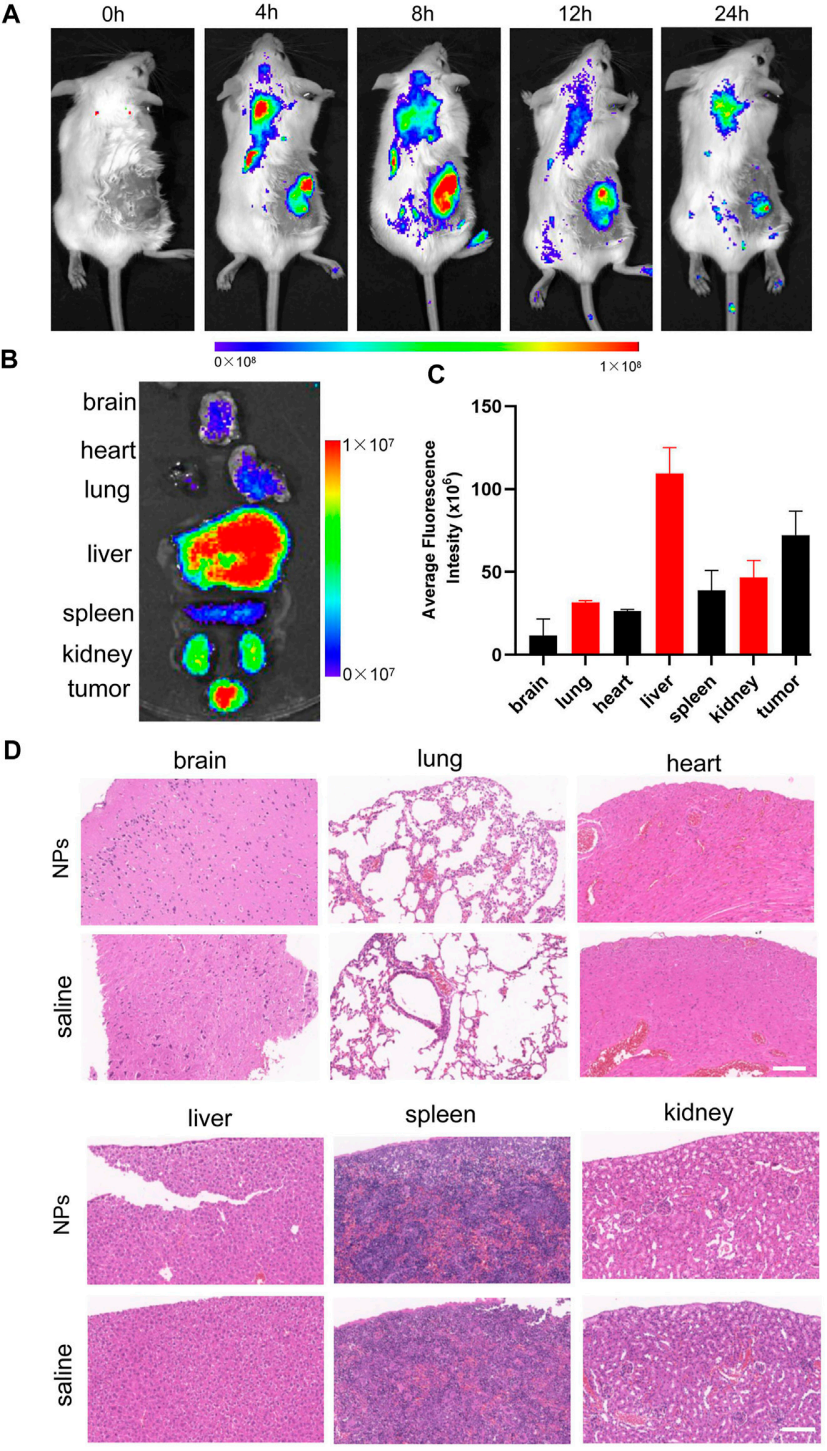
**(A)** Biodistribution of IR780/PLGA@MnO_2_ NPs in tumor-bearing mice at different time points by *in vivo* fluorescent imaging. **(B,C)** Fluorescence imaging of major organs and tumor at 8 h after injection of the IR780/PLGA@MnO_2_ NPs. **(D)** H&E-staining images of major organs collected from the IR780/PLGA@MnO_2_ NPs and saline groups.

To evaluate the biodistribution of IR780/PLGA@MnO_2_ NPs, the *in vivo* fluorescent imaging at different time points were carried out. As shown in Figure 6A, before injection of the IR780/PLGA@MnO_2_ NPs, there were no red fluorescent signals in the tumor region of tumor bearing mice. Obvious fluorescence was observed at the tumor site after injection 4 h, arrived to the peak intensity at 8 h and maintained until 24 h. The fluorescence of the major organs and tumor after injection 8 h was shown in Figures 6B,C, the IR780/PLGA@MnO_2_ NPs was mainly accumulated in the tumors and the reticuloendothelial system (such as liver), which was consistent with the previous studies (Chen et al., 2020).

To further assess the biotoxicity of IR780/PLGA@MnO_2_ NPs *in vivo*, the H&E stain of main organs was observed after 14 days injection. As shown in Figure 6D, there was no apparent changes in pathological damage and inflammation lesion in the major organs (brain, heart, liver, spleen, lung, and kidney) with injection of IR780/PLGA@MnO_2_ NPs, which proved that IR780/PLGA@MnO_2_ NPs have low potential toxicity and was biosafe *in vivo* for further tumor SDT.

### MR imaging in vitro and in vivo

**FIGURE 7:**
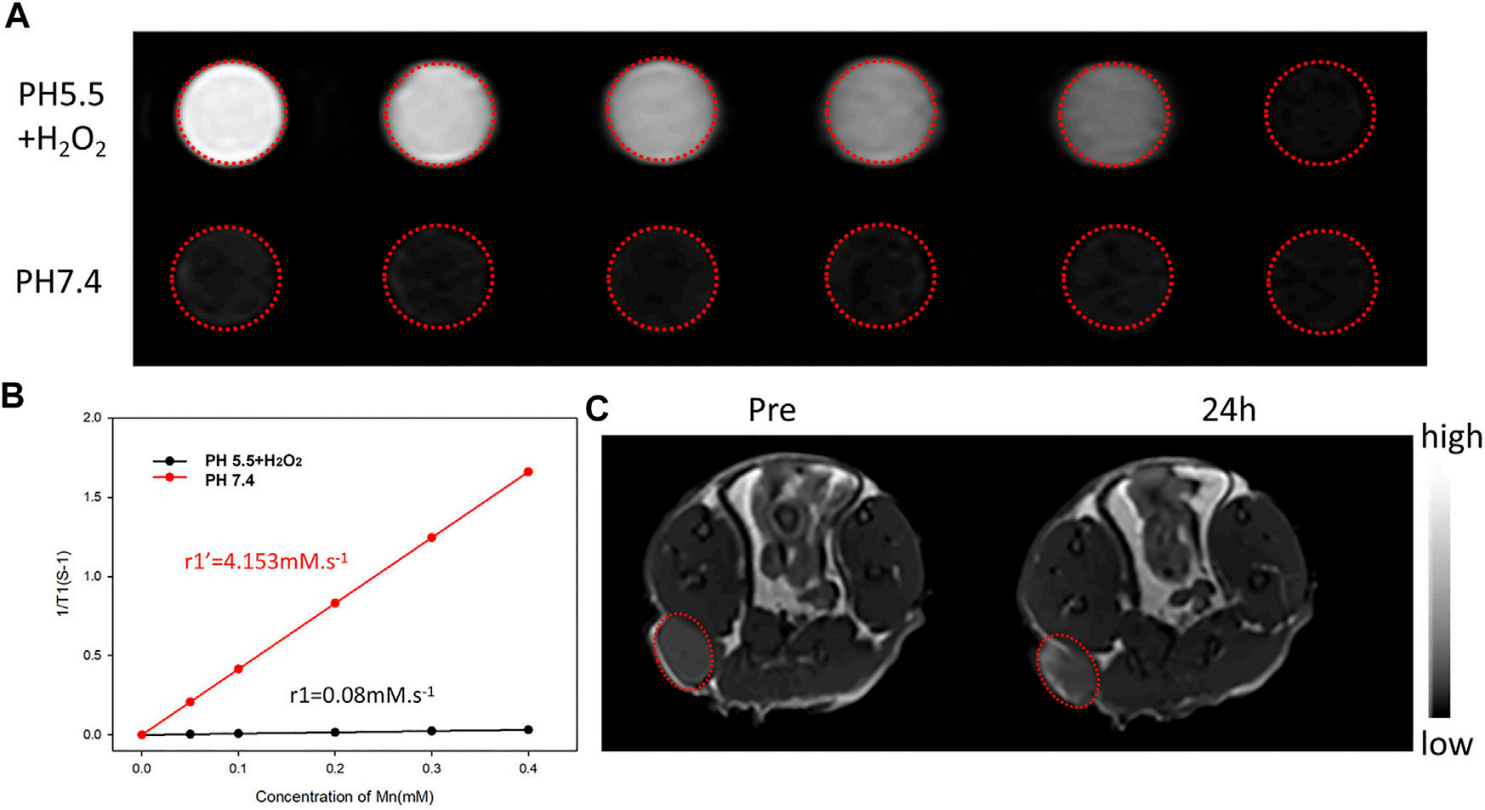
**(A)** T1-weighted MR images of IR780/PLGA@MnO_2_ NPs suspensions at different Mn concentrations and pH values with H_2_O_2_. **(B)** The longitudinal (r1) relaxation rate *vs.* the concentration of the IR780/PLGA@MnO_2_ NPs (in terms of Mn). **(C)** *In vivo* MR images of mice bearing implanted 4T1 cancer before and after 24 h of the intravenous injection of IR780/PLGA@MnO_2_ NPs.

As we known, because of high spatial resolution and deep tissue penetration, MRI is widely used as an imaging modality in clinical diagnosis. Several manganese dioxide related contrast agents that respond to tumor microenvironment, such as low-pH and excess-H_2_O_2_, have been reported for enhancement MR imaging. The relaxation properties of IR780/PLGA@MnO_2_ NPs under different buffer *in vitro* were verified to investigate the MR imaging performance using a 3.0 T clinical MRI scanner. As shown in Figure 7A, in the presence of acid (pH 5.5) and H_2_O_2_, IR780/PLGA@MnO_2_ NPs shows that the brightness of the T1 weighted MR images increased rapidly with the increasing MnO_2_ concentration. As the MnO_2_ concentration increased, the T1 weighted MR images showed brighter. However, in the presence of the physiological environment pH 7.4, the change of the brightness was small and the increase of the T1 weighted MR images was relatively weaker. Moreover, as shown in Figure 7B, the T1 relaxation coefficient (r1) is also increased from 0.08 × 10^–3^ m/s (pH 7.4) to 4.1 × 10^–3^ m/s (pH 5.0 and H_2_O_2_), which demonstrated that IR780/PLGA@MnO_2_ NPs can be used as tumor microenvironment stimulate-responsive T1 MRI contrast agents.

Then the T1-weighted MRI of the tumor before and after 24 h injection of IR780/PLGA@MnO_2_ NPs *in vivo* was investigated. As shown in Figure 7C, compared with before injection, the tumor area turned bright after injection of IR780/PLGA@MnO_2_ NPs and the signal intensity (SI) in the tumor area obviously increased at 24 h after injection, which indicated that IR780/PLGA@MnO_2_ NPs can accumulate at the tumor site and have the capability of tumor microenvironment -activated MR imaging.

### *In vivo* tumor hypoxia environment after different treatments

**FIGURE 8:**
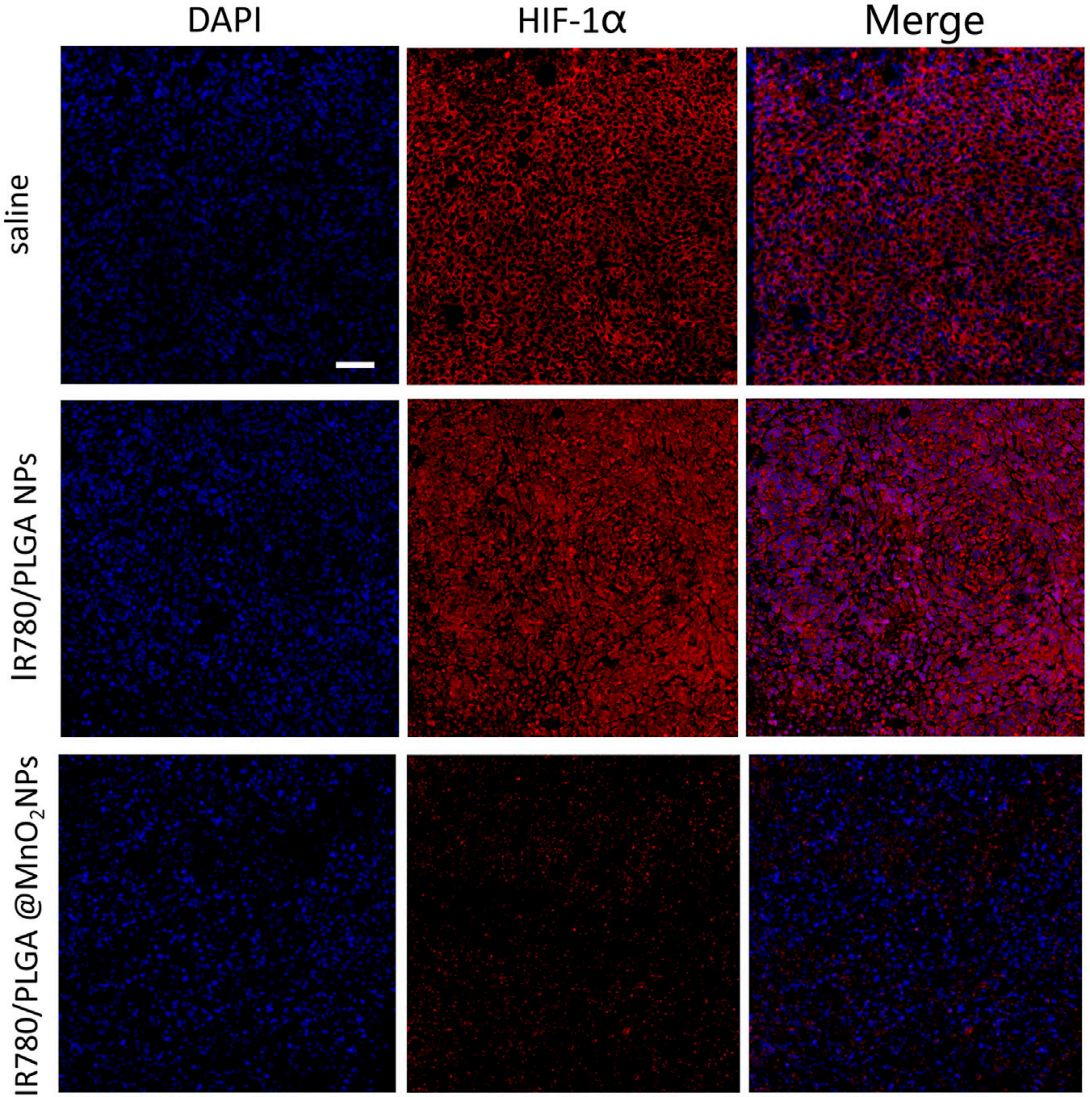
Representative immunofluorescence images of tumor slices stained by the HIF-1α under different treatments (scale bar, 200 µm).

Tumor hypoxia plays an important role in SDT resistance, angiogenesis and invasiveness of tumor, the hypoxic tumor microenvironment highly express hypoxia-inducible factors (HIFs). In this study, the capability of O_2_ production *via* IR780/PLGA@MnO_2_ NPs to relieve tumor hypoxia *in vitro* were confirmed. Then, *in vivo*, a HIF-1α probe was used to verify the tumor hypoxia with different treatments by immunofluorescence assay. As shown in Figure 8, there was strong red fluorescence in the saline and IR780/PLGA NPs groups, which suggested obvious hypoxia tumor conditions. Comparatively, the red fluorescence of the group treated with IR780/PLGA@MnO_2_ NPs was much weaker, which demonstrated that tumor hypoxia was relieved by IR780/PLGA@MnO_2_ NPs due to O_2_ production by MnO_2_.

### Antitumor efficacy *in vivo*

**FIGURE 9:**
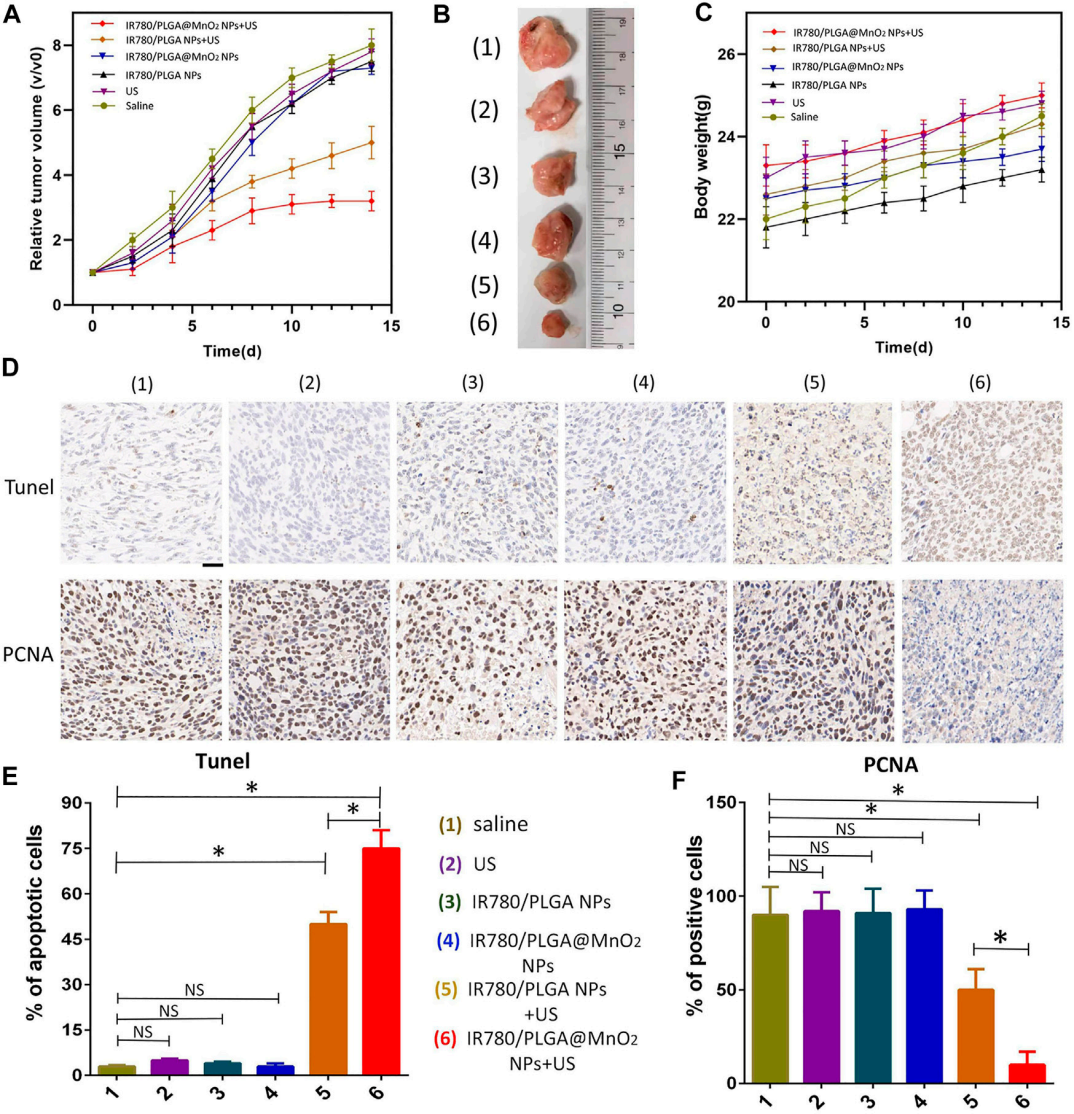
**(A)** The relative tumor growth curves and **(B)** photographs of tumors during the various treatments after 14 days. **(C)** The weight growth curves of different groups of mice after various treatments. **(D)** Microscopy images of TUNEL and PCNA assays of stained tumor tissues at different treatments: (Hussain and Nguyen, 2014) Saline, (Wells, 2006) US, (Wu et al., 2005) IR780/PLGA NPs, (Ahmadi et al., 2020) IR780/PLGA@MnO_2_NPs, (Qian et al., 2016) IR780/PLGA NPs + US, (Wood and Sehgal, 2015) IR780/PLGA@MnO_2_ NPs + US (scale bar, 200 µm). **(E)** The relative quantification of TUNEL and **(F)** PCNA after different treatments. (*p < 0.05).

The effectiveness of SDT therapy of the IR780/PLGA@MnO_2_ NPs *in vivo* were investigated. There were 6 groups with different treatments: (Hussain and Nguyen, 2014) saline, (Wells, 2006) US irradiation, (Wu et al., 2005) IR780/PLGA NPs, (Ahmadi et al., 2020) IR780/PLGA@MnO_2_ NPs, (Qian et al., 2016) IR780/PLGA NPs + US, (Wood and Sehgal, 2015) IR780/PLGA@MnO_2_ NPs + US. As shown in Figure 9A, after 14 days of treatments, the tumor volumes increased rapidly in the saline, US irradiation, IR780/PLGA@MnO_2_ NPs groups with 6-, 6.5-, 7-fold increase of the average tumor volumes, respectively. The tumor volume increased 5.5-fold in the group of IR780/PLGA NPs and US irradiation. However, the growth of tumors is slowly increased in the group of IR780/PLGA@MnO_2_ NPs and US irradiation. Figure 9B showed photographs of tumors during the various treatments after 14 days, indicating that the IR780/PLGA@MnO_2_ NPs combined with US irradiation could inhibit tumor growth through SDT and that the effectiveness against tumor of IR780/PLGA NPs coated with MnO_2_ was better than the IR780/PLGA NPs without MnO_2_. As shown in Figure 9C, there was bare weight loss of the mice in the 6 groups, which implied the good biosafety of PLGA/IR780@MnO_2_ NPs and US. The therapeutic efficacy of SDT was further evaluated by the TUNEL and PCNA assays of the tumor tissues. As shown in Figures 9D,E, compared with the groups of (Hussain and Nguyen, 2014) saline, (Wells, 2006) US irradiation, (Wu et al., 2005) IR780/PLGA NPs and (Ahmadi et al., 2020) IR780/PLGA@MnO_2_ NPs, the proliferative cells with brown nuclear staining of the TUNEL assay of the (Qian et al., 2016) IR780/PLGA NPs + US group was higher, but they were lower than that in the (Wood and Sehgal, 2015) IR780/PLGA@MnO_2_ NPs + US group. Opposite to the TUNEL expression pattern, the proliferative cells with brown nuclear staining of PCNA expression were the lowest in the (Wood and Sehgal, 2015) IR780/PLGA@MnO_2_ NPs + US group (Figures 9D,F). These results indicated that the PH-responsive and H_2_O_2_-triggered SDT based on the IR780/PLGA@MnO_2_NPs is an effective platform to inhibit tumor growth with negligible systemic toxicity.

## Conclusion

In summary, we successfully developed a multi-layer core-shell nanostructure of IR780/PLGA@MnO_2_ NPs with a high IR780-loading and low-leaking, which enhanced the SDT effect of 4T1 tumor bearing mice by deep penetration, H_2_O_2_-sensitive oxygen supply, under the guidance of MRI imaging. Introduction of the MnO_2_ shell can be react with overexpressed acidic H_2_O_2_ of tumor site to product Mn^2+^ for MRI and generate O_2_ for the improvement of hypoxia for enhanced SDT. We believe that our design of the IR780/PLGA@MnO_2_ NPs as a tumor microenvironment-sensitive nanoplatform provides a promising strategy for cancer diagnosis and SDT treatment, which could be further applied for cancer theranostics.

## Data Availability

The original contributions presented in the study are included in the article/supplementary material, further inquiries can be directed to the corresponding author.
